# Challenges and opportunities in 2D materials for high-performance aqueous ammonium ion batteries

**DOI:** 10.1093/nsr/nwae433

**Published:** 2024-11-28

**Authors:** Jie Xu, Tao Liu, Xusheng Dong, Xiaoyi Dong, Wanhai Zhou, Xiaojie Li, Dongliang Chao, Zhen Zhou, Ruizheng Zhao

**Affiliations:** Engineering Research Center of Advanced Functional Material Manufacturing of Ministry of Education, School of Chemical Engineering, Zhengzhou University, Zhengzhou 450001, China; Engineering Research Center of Advanced Functional Material Manufacturing of Ministry of Education, School of Chemical Engineering, Zhengzhou University, Zhengzhou 450001, China; Engineering Research Center of Advanced Functional Material Manufacturing of Ministry of Education, School of Chemical Engineering, Zhengzhou University, Zhengzhou 450001, China; Engineering Research Center of Advanced Functional Material Manufacturing of Ministry of Education, School of Chemical Engineering, Zhengzhou University, Zhengzhou 450001, China; Laboratory of Advanced Materials, Shanghai Key Laboratory of Molecular Catalysis and Innovative Materials, State Key Laboratory of Molecular Engineering of Polymers and School of Chemistry and Materials, Fudan University, Shanghai 200433, China; Engineering Research Center of Advanced Functional Material Manufacturing of Ministry of Education, School of Chemical Engineering, Zhengzhou University, Zhengzhou 450001, China; Laboratory of Advanced Materials, Shanghai Key Laboratory of Molecular Catalysis and Innovative Materials, State Key Laboratory of Molecular Engineering of Polymers and School of Chemistry and Materials, Fudan University, Shanghai 200433, China; Engineering Research Center of Advanced Functional Material Manufacturing of Ministry of Education, School of Chemical Engineering, Zhengzhou University, Zhengzhou 450001, China; Engineering Research Center of Advanced Functional Material Manufacturing of Ministry of Education, School of Chemical Engineering, Zhengzhou University, Zhengzhou 450001, China; State Key Laboratory of Inorganic Synthesis and Preparative Chemistry, College of Chemistry, Jilin University, Changchun 130012, China

**Keywords:** aqueous ammonium ion batteries, non-metallic charge carriers, 2D electrode materials, storage mechanisms, control strategies

## Abstract

Aqueous ammonium ion batteries (AAIBs) have attracted considerable attention due to their high safety and rapid diffusion kinetics. Unlike spherical metal ions, NH_4_^+^ forms hydrogen bonds with host materials, leading to a unique storage mechanism. A variety of electrode materials have been proposed for AAIBs, but their performance often falls short in terms of future energy storage needs. Hence, there is a critical need to design and develop advanced electrode materials for AAIBs. 2D materials, with their tunable interlayer spacing, remarkable interfacial chemistry and abundant surface functional groups, are an ideal choice for electrode materials for NH_4_^+^ storage. This review highlights the latest research on 2D electrode materials for AAIBs, providing insights into their working principles, NH_4_^+^ storage mechanisms and control strategies for designing high-performance AAIBs. Furthermore, a summary and future perspectives on 2D electrode materials in the development of AAIBs are provided, aiming to promote the advancement of high-performance AAIBs.

## Teaser

This review focuses on the NH4+ storage mechanisms and strategies of 2D materials in constructing high-performance aqueous ammonium ion batteries, which aims to promote their widespread application in the field of energy storage.

## INTRODUCTION

**Figure 1.**
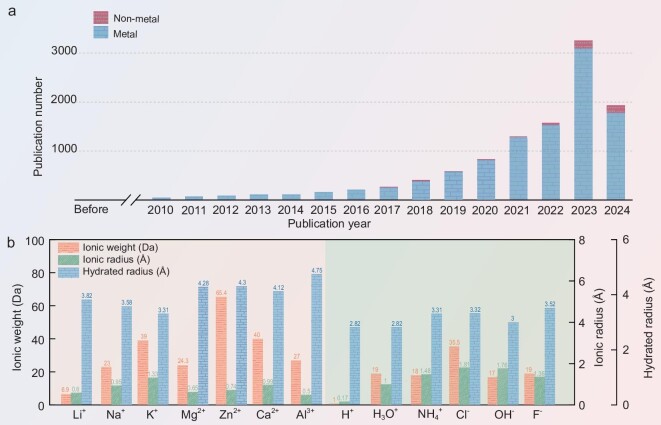
Current status and properties of ABs. (a) The number of publications for ABs corresponding to different carriers (data from Web of Science on 30 September 2024). (b) Comparison between the ionic weight, ionic radius and hydrated radius of certain metallic and non-metallic ions. Reproduced from ref. [12] with permission.

With the current shortage of fossil fuels and the limited large-scale application of intermittent energy sources such as wind, solar and tidal energy, there is an urgent need to develop new, efficient and long-lasting energy storage devices [1,2]. High-energy-density lithium-ion batteries have revolutionized many aspects of modern life, particularly in portable electronics and electric vehicles. However, the flammability of the organic electrolytes used in these batteries poses significant safety risks, such as thermal runaway, which can lead to fire or explosion and then limit their further development [3]. In contrast, aqueous batteries (ABs) with metal and non-metal carriers, which use an aqueous salt solution as the electrolyte, have become research hotspots due to their cost-effectiveness, intrinsically high safety and high ionic conductivity [4] (Fig. 1a). As the essential elements of ABs, the interaction between charge carriers and electrode materials is crucial for understanding the storage mechanism and electrochemical properties of batteries. In recent years, the research has mainly concentrated on metal ions as charge carriers due to their electrostatic interactions and relatively high energy density, such as Li^+^ [5], Na^+^ [6], K^+^ [7], Mg^2+^ [8], Zn^2+^ [9], Ca^2+^ [10] and Al^3+^ [11]. However, they suffer from poor cycle life due to their high molar mass, large hydration radius and high electrolyte corrosion, which limits their large-scale commercial application (Fig. 1b).

**Figure 2.**
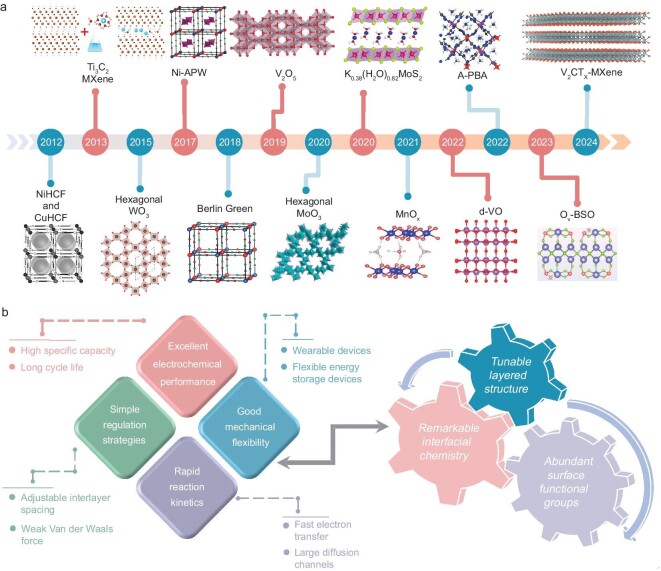
Status and advantages of host materials in AAIBs. (a) Roadmap with major achievements of AAIBs. (b) A schematic of the advantages of 2D materials.

In contrast, non-metallic ions are environmentally friendly, resourceful, sustainable and dendrite-free, which in turn makes non-metallic ion batteries highly competitive in the field of large-scale energy storage systems such as power grids, electric vehicles and energy storage in extreme environments. Aqueous ammonium ion batteries (AAIBs) with NH_4_^+^ as the charge carrier have received a lot of attention due to their resource abundance, widespread availability, small molar mass, fast diffusion kinetics, and the unique interaction between NH_4_^+^ and host materials (Fig. 1b) [12,13]. The working environment for AAIBs is relatively mild compared with other non-metallic ions, making them more competitive in extreme conditions. The pioneering research on AAIBs by Cui and his colleagues investigated the possibility of using Prussian blue analogues as electrodes for NH_4_^+^ storage, but the capacity provided by these materials is limited [14]. Since then, there has been increasing interest in researching electrode materials for AAIBs and significant achievements have been made in this field (Fig. 2a). Despite all the above advantages of AAIBs, the electrode material usually suffers from low specific capacity and poor structural stability due to the large ionic radius of NH_4_^+^ (1.48 Å) during the cycling process, which negatively affects its cycle stability. Additionally, the hydrogen evolution reaction and oxygen evolution reaction restrict the practical electrochemical stable window of AAIBs, accompanying pH sensitivity problem. Consequently, compared with the mainstream lithium-ion batteries, the main challenge of AAIBs is the insufficient energy density due to their low specific capacity and output voltage, which does not meet current demands. Researchers have made significant efforts to address these issues by designing high-performance electrode materials, regulating electrolytes and optimizing current collectors and separators [15–18]. In comparison, the design of high-performance electrode materials is one of the most important strategies to effectively enhance the performance of AAIBs, as these materials typically exhibit superior electronic and ionic conductivity. This, in turn, significantly increases the energy density, extends the cycle life and reduces the risk of thermal runaway, thereby improving the overall safety of the batteries.

Table 1 summarizes the properties of different host materials in AAIBs. Specifically, 1D materials are not conducive to achieving superior rate performance due to their low electrical conductivity and narrow ion diffusion pathways. 3D materials have large framework structures but suffer from poor structural stability, which has a negative impact on the cycle life of batteries. On the contrary, 2D materials, including graphene [19], transition metal oxides/dichalcogenides (TMOs/TMDs) [20,21], layered double hydroxides (LDHs) [22,23], transition metal carbides/nitrides, carbonitrides (MXenes) [24,25] and metal/covalent organic frameworks (MOFs/COFs) [26,27], are excellent candidates for NH_4_^+^ storage compared to other materials due to their tunable layer structure, remarkable interfacial chemistry and abundant surface functional groups. These features facilitate rapid reaction kinetics and outstanding electrochemical performance. Furthermore, 2D materials generally exhibit good mechanical flexibility, allowing them to withstand multiple bends and deformations without compromising performance, making them highly suitable for flexible and wearable energy storage devices. In summary, 2D materials offer significant advantages for AAIBs due to their large specific surface area, structural tunability, robust mechanical properties and enhanced ionic conductivity, positioning them as a focal point for research aimed at optimizing battery performance (Fig. 2b). In the most recent studies, Wu’s group prepared Bi_2_Se_3_ nanodots embedded in porous carbon nanofibers and investigated the material's performance in Zn^2+^ and NH_4_^+^ storage. The material exhibited a discharge capacity of up to 171 mAh g^−1^ even under a high loading condition of 18 mg cm^−2^ in AAIBs [28]. A 2D heteroligand-based copper-organic framework was developed by Xu's team. Thanks to the effective regulation of electron delocalization by the heteroligand and the inherent hydrogen bond cage mechanism among NH_4_^+^ ions, AAIBs show a high specific energy density of 211.84 Wh kg^−1^ [29]. Although some 2D materials have been utilized in AAIBs, the energy density of these batteries still falls short of expectations and needs further improvement. Meanwhile, the design of high-performance electrode materials requires a deep understanding of their energy storage mechanisms. However, most current reviews on AAIBs mainly focus on the types of electrode materials, with few comprehensive summaries of the storage mechanisms, especially for 2D materials applied in AAIBs [30,31]. Therefore, a summary of the energy storage mechanisms of 2D materials is needed to provide theoretical guidance and practical reference for researchers.

**Table 1.** Diffusion paths and properties of some materials in different dimensions for AAIBs.

Dimensionality	Channel type and size	Materials	Specific capacity (mAh g^−1^) @ current density (A g^−1^)	Capacity retention @ cycle number, current (A g^−1^)	Ref.
1D	Tunnel channel ∼ 0.50 × 0.50 nm	d-VO	200 @ 0.1	72.9% @ 1000, 2	[32]
		h-WO_3_	82 @ 1	68% @ 200 000, 20	[33]
		h-MoO_3_	115 @ 0.1	94%@ 100 000, 15	[34]
		VO_2_@C	300 @ 0.1	66.7% @ 1000, 2	[35]
2D	Interlayer space ∼ 0.3–0.96 nm	VS_2_/VO*_x_*	320 @ 0.1	43% @ 1000, 1	[36]
		E-CoNi DH	202 @ 0.6	83% @ 2000, 6	[37]
		MP-20	299 @ 1	96.3% @ 500, 2	[38]
		PTCDA/ Ti_3_C_2_T*_x_* MXene	202 @ 0.5	74% @ 10 000, 15	[39]
3D	Tunnel channel ∼ 0.32 × 0.32 nm	CuHCF	60 @ 0.05	91% @ 500, 0.5	[14]
		Fe[Fe(CN)_6_]_0.88_	90 @ 0.1	88% @ 450, 0.2	[40]
		Na-FeHCF	62 @ 0.25	≈100% @ 50 000, 2	[41]
		V_1.5_Fe(CN)_6_	93 @ 2	91.4% @ 2000, 2	[42]

This review aims to explore in depth the application of 2D materials in AAIBs. We provide a comprehensive introduction to various 2D materials and their roles in the energy storage process of AAIBs. Building on this foundation, we systematically summarize five major NH_4_^+^ storage mechanisms specific to 2D materials. To further improve the electrochemical performance of these materials, we focus on the various control strategies, including interlayer structure design, surface chemistry regulation, crystal water adjustment, amorphous structure regulation and heterostructure construction. These strategies provide a crucial theoretical basis for optimizing the application of 2D materials in AAIBs. Finally, the construction of high-performance AAIBs is thoroughly discussed from an overall perspective, with the aim of promoting the widespread application of AAIBs in the field of energy storage.

## WORKING PRINCIPLE OF AAIBS

**Figure 3.**
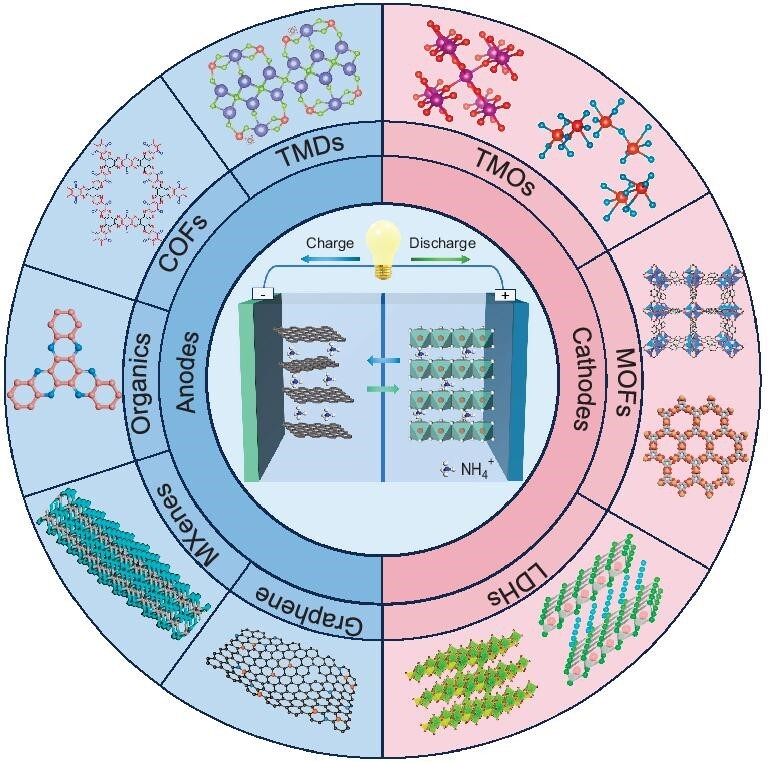
The working principle and the main 2D electrode materials of AAIBs.

During the discharge/charge process, NH_4_^+^ is inserted into and extracted from the electrode materials, similar to a conventional rocking chair metal battery. Specifically, during the charge process of AAIBs (Fig. 3), NH_4_^+^ is extracted from the ammonium-rich cathode materials, passes through the separator and electrolyte, and moves towards the anode. Meanwhile, electrons of equal charge move from the cathode to the anode via an external circuit. At the anode, the electrons and NH_4_^+^ react with the anode material, completing the charging process. This converts electrical power from external sources into chemical energy, which is stored in the battery through the formation of chemical bonds. During the discharge process, the reverse occurs. NH_4_^+^ and electrons are released from the anode, shuttle back to the cathode and combine with the cathode material. This completes the discharge process, releasing the previously stored chemical energy as electrical energy to power external electronics.

Due to this reversibility, the electrode materials largely determine whether NH_4_^+^ can effectively diffuse and undergo reversible insertion/extraction, which in turn impacts the electrochemical performance of AAIBs. Therefore, a thorough understanding of the physicochemical properties of the host materials through various advanced methods is essential for the selection of optimal electrode materials. For NH_4_^+^ storage, the research on 2D materials as electrodes has made remarkable progress, demonstrating their good electrochemical properties, as shown in Fig. 3. Currently, cathode materials are crucial in determining the capacity and cycle life of batteries. These include TMOs/TMDs [43,44], LDHs [37,45] and MOFs [46], which offer the highest operating potentials. For example, recently, a reduced graphene oxide (rGO) layer was electrochemically deposited on MnO*_x_* [47]. Taking advantage of three functions of rGO: rapid electron transport, inhibition of Mn^2+^ diffusion, promotion of Mn^2+^ and NO*_x_*^−^ adsorption, rGO_60_/MnO*_x_* exhibited a high discharge capacity of 109 mAh g^−1^ at 5 A g^−1^ and good cyclic stability, with 92.6% after 1000 cycles. Although various cathode materials have demonstrated promising NH_4_^+^ storage capabilities, AAIBs are still in the early stages of development and exhibit inadequate energy densities and longevity. Further enhancements are needed, particularly for the anode materials, which primarily consist of TMDs [32,48], COFs [49], organic compounds [50,51], MXenes [52,53] and graphene [54]. For example, Zhang *et al*. [53] fabricated an anode using a ball-flower morphology MoS_2_ material anchored on MXene nanoflakes, which showed excellent rate capability in AAIBs. To enhance the readers’ understanding and knowledge of 2D electrode materials, we further summarize the electrochemical performance of different 2D electrode materials for storage of NH_4_^+^ in Table 2. Although these materials have shown good performance in NH_4_^+^ storage, further optimization is required to meet future demands for high energy density and long life. This ongoing research is expanding opportunities in the field of new energy and bridging the gap between the theoretical comprehension of battery systems and their practical applications.

**Table 2.** Summary of 2D materials and their electrochemical properties for NH_4_^+^ storage.

Types	Materials	Electrolytes	Specific capacity (mAh g^−1^) @ current density (A g^−1^)	Capacity retention @ cycle number, current (A g^−1^)	Ref.
Cathodes	V_2_O_5_	0.5 M (NH_4_)_2_SO_4_	≈100 @ 0.1	80.1% @ 30 000, 5	[55]
	MnO*_x_*	0.5 M NH_4_Ac	176 @ 0.5	94.7% @ 10 000, 5	[56]
	rGO_60_/MnO*_x_*	0.5 M NH_4_Ac	173 @ 0.5	92.6% @ 1000, 5	[47]
	α-MnO_2_	1 M (NH_4_)_2_SO_4_	219 @ 0.1	95.4% @ 10 000, 1	[43]
	Mn_3_Al_1_-LDH	0.5 M (NH_4_)_2_SO_4_	183.7 @ 0.1	81% @ 400, 0.1	[45]
	FVO	0.5 M (NH_4_)_2_SO_4_	88.6 @ 1	61% @ 500, 5	[57]
	NH_4_V_4_O_10_	0.5 M NH_4_Cl	155.5 @ 0.05	78.5% @ 200, 0.1	[53]
	NVO	1 M NH_4_Cl/PVA	169 @ 0.5	71% @ 14 000, 0.5	[54]
	VOPO_4_·2H_2_O	2 M NH_4_OTf	154.6 @ 0.1	40.6% @ 100, 0.1	[44]
	Cu-HHB/I_2_	1 M (NH_4_)_2_SO_4_	111.7 mF cm^−2^ @ 0.4 mA cm^−2^	91% @ 10 000, 2 mA cm^−2^	[46]
Anodes	PTCDI	1.0 M (NH_4_)_2_SO_4_	119 @ 0.24	89% @ 400, 1.2	[50]
	Bi_2_SeO_5_	1 M NH_4_Cl	341.0 @ 0.3	86.7% @ 7000, 3	[58]
	VO*_x_*@PPy	0.5 M NH_4_Ac	195.36 @ 0.2	51% @ 100, 1	[59]
	SMO	2 M CH_3_COONH_4_	185 @ 0.5	44% @ 500, 0.5	[48]
	KMS	1 M (NH_4_)_2_SO_4_	50.74 @ 0.5	56% @ 50, 0.5	[60]
	V_2_CT*_x_* MXene	0.5 M NH_4_Ac	115.9 @ 1	100% @ 5000, 5	[61]
	QA-COF	0.5 m (NH_4_)_2_SO_4_	220.4 @ 0.5	90.7% @ 500, 0.5	[49]
	AC	1 M NH_4_Cl/PVA gel	137 F g^−1^ @ 0.5	–	[54]

## DIFFERENT NH_4_^+^ STORAGE MECHANISMS IN 2D MATERIALS

Unlike spherical metal ions that have no preferential orientation, NH_4_^+^ is tetrahedral, leading to different insertion and adsorption behaviors. Owing to its distinctive structural and electrochemical characteristics, NH_4_^+^ interacts significantly with a wide range of electrode materials. NH_4_^+^ displays an extensive array of topological chemical behaviors in 2D matrix materials, with charge transfer occurring through the formation and breaking of hydrogen bonds as NH_4_^+^ interfaces with the host. The mechanism underlying NH_4_^+^ storage is complex and warrants ongoing exploration. Currently, there is no systematic summary of the energy storage mechanisms for 2D materials, and the known energy storage mechanisms of AAIBs include the following main aspects.

### NH_4_^+^ insertion/extraction mechanism

**Figure 4.**
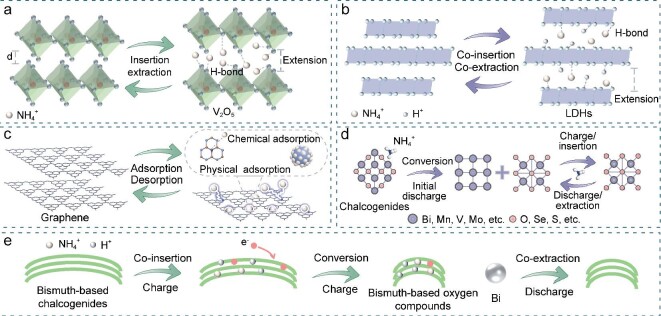
Illustration of energy storage mechanisms for 2D materials in AAIBs. Illustration of (a) NH_4_^+^ insertion/extraction mechanism, (b) NH_4_^+^/H^+^ co-insertion/extraction mechanism, (c) adsorption/desorption mechanism, (d) conversion reaction mechanism and (e) hybrid storage mechanism.

Among the various NH_4_^+^ storage materials, the most common storage mechanism involves the insertion/extraction of NH_4_^+^ within the material (Fig. 4a). NH_4_^+^ interacts with the atoms or molecules of the host material and facilitates electron transport, which has been observed in many practical applications. Specifically, during discharge, NH_4_^+^ is inserted into the material, increasing the interlayer spacing, whereas during charge, the process is reversed.

Some 2D materials with tunable structures can serve as highly efficient hosts for NH_4_^+^ insertion/extraction. For example, bi-layered V_2_O_5_ has been reported as an effective intercalation material for NH_4_^+^ [55,62]. Ji and collaborators [55] demonstrated that NH_4_^+^ can be reversibly inserted/extracted into bi-layered V_2_O_5_ by exploiting its unique pseudocapacitive behavior in ABs to achieve high storage capacity. By examining cyclic voltammetry (CV) curves in 0.5 M (NH_4_)_2_SO_4_ and H_2_SO_4_ solutions at the same pH value, they found that the current in the H_2_SO_4_ solution was only 2.2% of that in the (NH_4_)_2_SO_4_ electrolyte, indicating that the effect of H^+^ can be ignored. The energy storage mechanism was mainly due to NH_4_^+^ insertion/extraction. *Ex situ* analyses of Fourier transform infrared (FTIR) spectroscopy, Raman spectroscopy, solid-state nuclear magnetic resonance (NMR), X-ray diffraction (XRD) and density functional theory (DFT) calculations showed that NH_4_^+^ insertion/extraction in the host material was highly reversible, involving the breaking and reforming of hydrogen bonds during the cycle. A typical process is presented below, with the corresponding electrode reactions described by Equations (1) and (2). Previous studies concluded that NH_4_^+^ storage in layered VOPO_4_·2H_2_O was impossible because the removal of NH_4_^+^ from NH_4_VOPO_4_ inevitably leads to a phase change. However, Hu and co-workers [44] revised this understanding by demonstrating the highly reversible insertion/extraction behavior of NH_4_^+^ in a layered VOPO_4_·2H_2_O host, facilitated by the crystal water enhancement effect.

Discharge process:


(1) \begin{eqnarray*}
{{{\mathrm{V}}}_2}{{{\mathrm{O}}}_5} + x{\mathrm{NH}}_4^ + + x{{{\mathrm{e}}}^ - } \to ({{\mathrm{NH}}_4^ + } )_x {{{\mathrm{V}}}_2}{\mathrm{O}}_5^{x - }.
\end{eqnarray*}

Charge process:


(2) \begin{eqnarray*}
\left( {{{{\left. {{\mathrm{NH}}_4^ + } \right)}}_x}} \right.\!\! {{{\mathrm{V}}}_2}{\mathrm{O}}_5^{x - } \to {{{\mathrm{V}}}_2}{{{\mathrm{O}}}_5} + x{\mathrm{NH}}_4^ + + x{{{\mathrm{e}}}^ - }.
\end{eqnarray*}

Additionally, analogous mechanisms of energy storage and pathways of ion diffusion have been identified in Mn-based electrode materials for NH_4_^+^ storage [63,64]. Amorphous electrodeposited manganese oxide (MnO*_x_*) with a water structure has been verified as a promising NH_4_^+^ storage material, maintaining a stable structure after 40 cycles [56]. X-ray photoelectron spectroscopy (XPS) results indicated that the insertion effect of H^+^ was minimal and negligible, showing that H^+^ did not participate in the charge/discharge process. During the discharge, the insertion of NH_4_^+^ increased the lattice spacing. Characterizations and calculations indicated that the reversible NH_4_^+^ insertion/extraction involved hydrogen bond formation and fracture between NH_4_^+^ and MnO*_x_* layers. The combination of inorganic and organic materials is also an important direction for preparing NH_4_^+^ storage materials. Gao *et al*. [39] developed an anode material for AAIBs by combining Ti_3_C_2_T*_x_* MXene with perylene-3,4,9,10-tetracarboxylic dianhydride (PTCDA). This composite material demonstrated an ultra-long cycle life and excellent rate performance. *Ex-situ* XPS and scanning electron microscopy (SEM) were used to characterize the reversible NH_4_^+^ insertion/extraction during the charge/discharge process.

### NH_4_^+^/H^+^ co-insertion/extraction mechanism

Recently, researchers have developed materials with more defects and additional active sites, and based on this they proposed an innovative energy storage process, known as NH_4_^+^/H^+^ co-insertion. The rich vacancies in these materials improves their ionic conductivity and reduces the surface energy of materials, thereby facilitating proton insertion chemistry. Notably, some studies have demonstrated NH_4_^+^/H^+^ co-insertion/extraction into host materials during the charge/discharge process, as shown in Fig. 4b. Given this storage mechanism, the electrolyte concentrations should be maintained at moderate levels, because there are fewer free water molecules in a highly concentrated electrolyte, which can hinder the co-insertion behavior, especially when the hydrogen-bonding network of water is extensively disrupted [65]. However, the examination of this energy storage mechanism is in its preliminary stages. Further studies are needed to determine whether NH_4_^+^/H^+^ co-insertion competes with NH_4_^+^ for active sites or provides additional capacity.

Spectroscopic studies revealed a reversible NH_4_^+^/H^+^ co-insertion mechanism throughout the charging and discharging cycles of vanadium oxide/polypyrrole (VO*_x_*@PPy) material fabricated by electrodeposition in NH_4_Ac electrolyte. [59]. Energy dispersive X-ray spectroscopy analysis showed that the content ratio of nitrogen/vanadium increased during charging, further proving the insertion mechanism of NH_4_^+^. Additionally, the content of V–O–H bonds increased in the charged state, indicating the insertion of H^+^. The presence of NH_4_^+^ within the lattice of VO*_x_* contributes positively to the enhancement of its structural stability. The basic co-insertion/extraction expressions are shown in Equations (3) and (4). Similarly, a porous and amorphous manganese phosphate material (MP-20) prepared by electrodeposition was used for aqueous NH_4_^+^ energy storage [38]. The O 1s spectrum indicated that changes in peak values were due to changes in Mn–OH bonds. Combined with other tests, this suggested that H^+^ was involved during the charge process of MP-20. The N 1s spectrum and FTIR results indicated that NH_4_^+^ was inserted into and extracted from MP-20. It was also estimated that the proportion of capacity attributable to H^+^ insertion is ∼30% of the cumulative capacity of the MP-20 material. Therefore, the co-insertion/extraction of NH_4_^+^/H^+^ occurred during the energy storage process of MP-20. Additionally, the synthesis of an amorphous-structured activated Ni-Co double hydroxide (A-NiCo-DH) was achieved via electrodeposition, introducing H-vacancies in the electrochemical activation stage to create extra cation intercalation sites [66]. In the FTIR spectrum, the insertion and extraction process of NH_4_^+^ during the discharge/charge phase is reversible, which can be verified by the variation of the peak intensity of the N–H at 1404 cm^−1^. The H^+^ insertion behavior was demonstrated by the O 1s spectrum, where the content ratio of metal–O–H to metal–O increased in the discharge process and reversibly decreased after charge. The absence of notable variations in the XRD pattern is indicative of the electrodes maintaining their original phase. These results confirm the NH_4_^+^/H^+^ co-insertion/extraction mechanism within the A-NiCo-DH electrode. Notably, introducing H vacancies results in the increased exposure of electronegative oxygen atoms, which in turn increases the number of active sites and enhances the affinity of the electrode surface for cation adsorption.

Discharge process:


(3) \begin{eqnarray*}
&&{\mathrm{V}}{{{\mathrm{O}}}_x}@{\mathrm{PPy}} + m{\mathrm{NH}}_4^ + + n{{{\mathrm{H}}}^ + } + \left( {m + n} \right){{{\mathrm{e}}}^ - } \to\\
&&\quad \quad \left( {{{{\left. {{{{\mathrm{H}}}^ + }} \right)}}_n}}\!\! \right.\left( {{{{\left. {{\mathrm{NH}}_4^ + } \right)}}_m}}\!\! \right.{\mathrm{V}}{{{\mathrm{O}}}_x}@{\mathrm{PP}}{{{\mathrm{y}}}^{\left( {m + n} \right) - }}.\\
\end{eqnarray*}

Charge process:


(4) \begin{eqnarray*}
&&\left( {{{{\left. {{{{\mathrm{H}}}^ + }} \right)}}_n}}\!\! \right.\left( {{{{\left. {{\mathrm{NH}}_4^ + } \right)}}_m}}\!\! \right.{\mathrm{V}}{{{\mathrm{O}}}_x}@{\mathrm{PP}}{{{\mathrm{y}}}^{\left( {m + n} \right) - }} \to\\
&&\quad \quad\!\! {\mathrm{V}}{{{\mathrm{O}}}_x}@{\mathrm{PPy}} + m{\mathrm{NH}}_4^ + + n{{{\mathrm{H}}}^ + } + \left( {m + n} \right){{{\mathrm{e}}}^ - }.\\
\end{eqnarray*}

### Adsorption/desorption mechanism

During the cycling process of AAIBs, NH_4_^+^ adsorption/desorption typically occurs in addition to NH_4_^+^ insertion/extraction, and NH_4_^+^/H^+^ co-insertion/extraction. This process, depicted in Fig. 4c, is non-selective and is characterized by the absence of electron transfer or chemical bond formation. The tendency of NH_4_^+^ adsorption/desorption is intricately related to the surface properties of the electrode material, including specific surface area and pore size distribution. Consequently, porous materials with a high specific surface area, low mass and excellent adsorption properties have an inherent advantage [54,67,68].

Meng’s group [54] utilized activated carbon (AC) as the anode for NH_4_^+^ storage. The CV curves of AC exhibit a rectangular shape, demonstrating its typical double-layer capacitance. The galvanostatic charge/discharge curves are a symmetrical triangular shape, further illustrating this characteristic. NH_4_^+^ storage in AC is realized via physical adsorption at the electrolyte–electrode interface, a process that involves electrostatic interactions and does not involve charge transfer. The main adsorption/desorption process for AC is shown in Equation (5). Besides, as electrostatic adsorption is common in a vast array of electrode materials, the overall capacitance of pseudocapacitive materials inevitably contains an electrical double-layer capacitor (EDLC), usually in the range of 5% to 10% [66,69,70]. Accordingly, the storage mechanism of lots of electrode materials may include adsorption/desorption reactions. Zhang *et al*. [53] synthesized a MoS_2_/MXene composite material via the hydrothermal method. The changes in the content of N, and the valence of Mo were related to the adsorption/desorption of NH_4_^+^ during the discharge/charge process. During the discharge process, NH_4_^+^ was adsorbed on the surface of MoS_2_/MXene, resulting in the reduction of Mo.


(5) \begin{eqnarray*}
{\mathrm{AC}} + x{\mathrm{NH}}_4^ + \mathop \leftrightarrow \limits^{{\mathrm{NH}}_4^ + {\mathrm{\ adsorption}}/{\mathrm{desorption}}} \left( {{{{\left. {{\mathrm{NH}}_4^ + } \right)}}_x}} \right.\!\!{\mathrm{AC}}.
\end{eqnarray*}

For EDLC-based electrode materials, surface adsorption interactions are essential for energy storage. An increase in active sites on the electrode surface can enhance ion adsorption, thereby boosting the energy storage capacity of the material. The Brunauer-Emmett-Teller theory serves as a valuable tool for assessing the specific surface area of EDLC materials, which is instrumental in the design and fabrication of high-performance AAIBs.

### Conversion reaction mechanism

Unlike the ion insertion/extraction and adsorption/desorption chemistry that can be kinetically hindered, direct charge transfer exhibits a strong kinetic tendency. Organic molecules with distinctive functional groups, including conjugated C=O and C=N, have proven to be effective in storing NH_4_^+^ through chemical reactions. Specifically, during NH_4_^+^ insertion, the C=O group can undergo a reversible transformation through an enolization reaction, shifting its configuration from –C=O to –C–O–NH_4_^+^, which leads to the establishment of the C–O–NH_4_^+^ bond. Hydrogen bonds, known for their strong intermolecular force, have a pronounced effect on the adsorption properties of functional groups in organic substances. This creates flexible mechanisms for NH_4_^+^ storage, as opposed to rigid metal bonds, thereby improving rate capability. For example, in 2017, Ji *et al*. [50] constructed the first rocking chair AAIBs and studied the storage mechanism of 3,4,9,10-perylenetetracarboxylic diimide (PTCDI) with C=O functional groups as the anode electrode. PTCDI displayed two pairs of distinct redox peaks in the CV curve, which can be attributed to the reversible enolization reaction of the C=O group in PTCDI, which can combine with two NH_4_^+^. In addition, Tian and his co-workers [49] studied the unique intercalation chemistry of NH_4_^+^ on the COF based on a monomer containing quinone carbonyl oxygen and pyrazine nitrogen. Through a series of experimental characterizations under different charge and discharge conditions and calculations, it was shown that during the NH_4_^+^ insertion/extraction process, reversible transformations between C=O and C–O, as well as C=N and C–N, occur. NH_4_^+^ bridges the carbonyl O and pyrazine N atoms via H bonds (N−H···O and N−H···N), which chemically induces NH_4_^+^ to form more stable thermodynamic discharge products, thus achieving good electrochemical properties (220.4 mAh g^−1^ at a current density of 0.5 A g^−1^). A similar reaction process was observed in MXene-integrated perylene by Gao *et al*. [39]. In their study, O 1s and N 1s spectra revealed the existence of O–N bonds and the reversible change of C–O–NH_4_ bonds during the charge and discharge process, indicating that the reversible enolization occurred after NH_4_^+^ insertion into the host material.

In addition to the enolization reaction, other conversion reaction mechanisms are illustrated in Fig. 4d. During the first ion insertion process, certain electrode materials transform into new compounds that remain stable after recharge and do not revert to their original form. Subsequent charge and discharge cycles rely on the newly established structure, which allows for the reversible insertion/extraction of NH_4_^+^. This indicates that the transformation is semi-reversible in the first cycle. For example, the MnAl layered double hydroxide (Mn_3_Al_1_-LDH) studied by Hu *et al*. [45] for NH_4_^+^ storage demonstrated such a conversion reaction (Equations (6–8)), and it can be illustrated as follows. By analyzing the *ex-situ* XRD patterns in different charge and discharge states, it was observed that a new substance, identified as Mn_*x*_(OH)_*y*_SO_4_·5H_2_O, had formed and transitioned to an amorphous phase. This amorphous phase was well preserved in the subsequent cycles, indicating that the material changed from a crystalline to an amorphous phase during the NH_4_^+^ storage process.

The first cycle charge process:


(6) \begin{eqnarray*}
&&{\mathrm{M}}{{{\mathrm{n}}}^{2 + }} ( {{\mathrm{in\ crystalline\ LDH}}} ) \to {\mathrm{M}}{{{\mathrm{n}}}^{4 + }}\\
&&\quad\quad ( {{\mathrm{in\ amorphous\ state}}} ) + 2{{{\mathrm{e}}}^ - }.
\end{eqnarray*}

The subsequent cycles:

Discharge process:


(7) \begin{eqnarray*}
&&{\mathrm{M}}{{{\mathrm{n}}}^{4 + }}\left( {{\mathrm{in\ amorphous\ state}}} \right) + {{{\mathrm{e}}}^ - }\mathop \to \limits^{{\mathrm{NH}}_4^ + {\mathrm{\ insertion}}}\\
&&\quad\quad {\mathrm{M}}{{{\mathrm{n}}}^{3 + }}\left( {{\mathrm{in\ amorphous\ state}}} \right).
\end{eqnarray*}

Charge process:


(8) \begin{eqnarray*}
&&{\mathrm{M}}{{{\mathrm{n}}}^{3 + }}\left( {{\mathrm{in\ amorphous\ state}}} \right)\mathop \to \limits^{{\mathrm{NH}}_4^ + {\mathrm{\ extraction}}}\\
&&\quad\quad {\mathrm{M}}{{{\mathrm{n}}}^{4 + }} + {{{\mathrm{e}}}^ - }\left( {{\mathrm{in\ amorphous\ state}}} \right).
\end{eqnarray*}

### Hybrid storage mechanism

In addition to the single NH_4_^+^ storage mechanism described above, multiple storage mechanisms typically occur in some materials (Fig. 4e). For example, Meng *et al*. [54] reported an ammonium vanadium oxide (NVO) framework in NH_4_Cl/polyvinyl alcohol (PVA) gel electrolyte with a mesoporous structure and a specific surface area of ∼270 m^2^ g^−1^, signifying the critical involvement of double-layer capacitance in the energy storage performance of NVO nanosheets. The CV curve presented a quasi-rectangular shape and two pairs of broad peaks, which were attributed to the double-layer capacitance and hydrogen bond formed by NH_4_^+^ insertion into the host material. These results demonstrated that the storage mechanisms of NVO included NH_4_^+^ insertion/extraction and adsorption/desorption. The group [67] also investigated the intercalation of polyaniline as a guest molecule into hydrated vanadium oxide to form PVO and observed similar phenomena. Additionally, Zhang *et al*. [53] synthesized a MoS_2_/MXene composite material via a hydrothermal method and found that changes in the content of N and the valence state of Mo were related to the adsorption/desorption of NH_4_^+^ during the discharge/charge process. A similar process of hybrid storage mechanism was observed in porous Bi_2_SeO_5_ with high oxygen vacancies (O_v_-BSO) by Wang *et al*. [58]. *Ex-situ* XRD and FTIR analysis revealed changes in characteristic peaks including the N–H peak, which were attributed to the reversible insertion/extraction of NH_4_^+^. CV curves of O_v_-BSO tested in NH_4_Cl/DMSO and H_2_SO_4_ electrolytes at the same pH showed a larger capacity in the H_2_SO_4_ electrolyte, indicating the co-insertion/extraction mechanism of NH_4_^+^/H^+^. Additionally, the appearance and disappearance of characteristic peaks of new species in the XRD pattern, along with the presence of Se^2−^ and Cl^−^ signals in the XPS pattern, confirmed that a conversion reaction had occurred. Specifically, O_v_-BSO generated BiSe and Bi during the discharge process and BiOCl during the charge process. Therefore, the electrochemical behavior in layered O_v_-BSO is characterized by a reversible NH_4_^+^/H^+^ co-insertion/extraction mechanism, which is intertwined with the generation and breaking of hydrogen bonds, as well as a conversion reaction. Notably, the introduction of oxygen vacancies significantly boosts the transfer of ions and electrons, and also promotes the establishment of hydrogen bonds between NH_4_^+^ and the host material.

Furthermore, Sun *et al*. [61] investigated the NH_4_^+^ storage behavior of V_2_CT*_x_* MXene in NH_4_Ac electrolyte and discovered an enhancement effect attributed to Ac^−^. The storage mechanism of NH_4_^+^ was explored by *in-situ* electrochemical quartz crystal microbalance, revealing a two-step electrochemical process (Equations (9–12)) that can be illustrated as follows. The first step involved the electrostatic adsorption/desorption of NH_4_^+^ on the surface of MXene. In the second step, a redox reaction occurred between d-V_2_CT*_x_* and the [NH_4_^+^(HAc)_3_] group, where the central NH_4_^+^ acted as a pseudoproton, facilitating the transition of the terminal groups from –O to –O···HN, and thereby changing the valence state of V. Through these two steps, d-V_2_CT*_x_* MXene demonstrated high reversible capacity and excellent cyclic stability.

Discharging process:


(9) \begin{eqnarray*}
&&{{{\mathrm{V}}}_2}{\mathrm{C}}{{{\mathrm{T}}}_x} + {\mathrm{NH}}_4^ + + {{{\mathrm{e}}}^ - } \to {{{\mathrm{V}}}_2}{\mathrm{C}}{{{\mathrm{T}}}_x} \cdot \left( {{{{\mathrm{e}}}^ - }} \right) \cdot \left[ {{\mathrm{NH}}_4^ + } \right]\\
&&\quad\quad \left( {{\mathrm{adsorption}},{\mathrm{\ EDLC}}}\right);
\end{eqnarray*}

(10) \begin{eqnarray*}
&&{{{\mathrm{V}}}_2}{\mathrm{C}}{{{\mathrm{T}}}_x} + {\mathrm{NH}}_4^ + \left( {{{{\left. {{\mathrm{HAc}}} \right)}}_3}} \right. + {{{\mathrm{e}}}^ - } \to \left( {{\mathrm{V}},} \right.{{\left. {{{{\mathrm{e}}}^ - }} \right)}_2} {\mathrm{C}}{{{\mathrm{T}}}_x} \\
&&\quad \cdot \left[ {{\mathrm{NH}}_4^ + \left( {{{{\left. {{\mathrm{HAc}}} \right)}}_3}} \right.} \right]{\mathrm{\ }}\left( {{\mathrm{reduction}},{\mathrm{\ pseudocapacity}}} \right).\\
\end{eqnarray*}

Charging process:


(11) \begin{eqnarray*}
&&{{{\mathrm{V}}}_2}{\mathrm{C}}{{{\mathrm{T}}}_x} \cdot \left( {{{{\mathrm{e}}}^ - }} \right) \cdot \left[ {{\mathrm{NH}}_4^ + } \right] \to {{{\mathrm{V}}}_2}{\mathrm{C}}{{{\mathrm{T}}}_x}\\
&&\quad +\ {\mathrm{NH}}_4^ + + {{{\mathrm{e}}}^ - }{\mathrm{\ }}\left( {{\mathrm{desorption}}} \right);
\end{eqnarray*}

(12) \begin{eqnarray*}
&&\left( {{\mathrm{V}},} \right.{{\left. {{{{\mathrm{e}}}^ - }} \right)}_2}{\mathrm{C}}{{{\mathrm{T}}}_x} \cdot \left[ {{\mathrm{NH}}_4^ + \left( {{{{\left. {{\mathrm{HAc}}} \right)}}_3}} \right.} \right] \to {{{\mathrm{V}}}_2}{\mathrm{C}}{{{\mathrm{T}}}_x}\\
&& +\ {\mathrm{NH}}_4^ + \left( {{{{\left. {{\mathrm{HAc}}} \right)}}_3}} \right. + {{{\mathrm{e}}}^ - }{\mathrm{\ }}\left( {{\mathrm{oxidation}},{\mathrm{\ pseudocapacity}}} \right).\\
\end{eqnarray*}

## STRATEGIES FOR CONSTRUCTING HIGH-PERFORMANCE AAIBS USING 2D MATERIALS

Properties of 2D electrode materials, such as high energy density and long cycle life, are pivotal for superior performance. The pursuit of innovative 2D electrode materials characterized by elevated specific capacity, robust structural stability and distinguished rate performance is continually at the forefront of the development of AAIBs. As the research progresses, it becomes apparent that directly obtained 2D electrode materials often exhibit certain deficiencies, such as small interlayer spacing, re-stacking and surface defects, which significantly affect their electrochemical performance. To overcome these obstacles, we present a systematic summary of the existing research, focusing on the structural properties of 2D materials, and propose constructive insights for strategic design methods. These strategies include interlayer structure design, surface chemistry regulation, crystal water adjustment, amorphous structure regulation and heterostructure construction. Addressing these factors is essential for future profound analysis and development.

### Interlayer structure design

#### Interlayer spacing optimization

**Figure 5.**
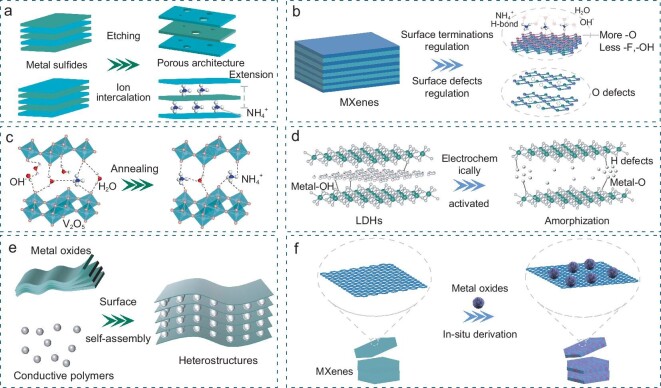
Schematic illustration of strategies for 2D materials in the construction of high-performance AAIBs. (a) Interlayer structure design. (b) Surface chemistry regulation. (c) Crystal water adjustment. (d) Amorphous structure regulation. Heterostructure construction of (e) surface self-assembly strategy and (f) *in-situ* derivation strategy.

Although NH_4_^+^ has a small hydrated ionic radius, its large ionic radius requires suitable electrode materials. 2D materials, with easily adjustable interlayer spacing, are ideal for NH_4_^+^ energy storage. The schematic representation of the interlayer design is shown in Fig. 5a. Based on the suitable layer spacing and hydrophilic properties of Ti_3_C_2_T*_x_*, Gogotsi’s group pioneered the insertion of NH_4_^+^ into the interlayers of Ti_3_C_2_T*_x_*, resulting in a capacitance value of over 300 F cm^−2^, which significantly outperforms porous carbon [71]. Chen's group [55] annealed bi-layered V_2_O_5_ at 80°C, 300°C and 400°C and designated these as VO80, VO300 and VO400. Electrochemical tests revealed that VO300 had significantly better performance than VO80 and VO400, which can be attributed to its larger interlayer spacing, which enables ultra-fast NH_4_^+^ storage. Moreover, NH_4_V_4_O_10_, with its large interlayer spacing and layered structure, serves as an intrinsic NH_4_^+^ source suitable for cathodes in NH_4_^+^ storage [72]. NH_4_V_4_O_10_ anchored in the carbon fiber (CF@NH_4_V_4_O_10_) was used as the electrode material of AAIBs [72]. XRD and high-resolution transmission electron microscopy (HRTEM) images confirmed that NH_4_V_4_O_10_ has a larger d-spacing of 9.6 Å. Due to its large interlayer spacing, CF@NH_4_V_4_O_10_ exhibited a high specific capacity of 104 mAh g^−1^ and excellent rate performance.

Expanding the interlayer spacing by molecular or ionic pre-intercalation is another effective strategy [73]. Huang *et al*. [60] successfully intercalated hydrated K^+^ into MoS_2_ to produce KMS (K_0.38_(H_2_O)_0.82_MoS_2_), which, in particular, expanded the interlayer spacing from 6.2 Å to 9.3 Å. The expanded interlayer spacing enhanced the ion diffusion capability of KMS, making it a promising host material for the storage of multiple cations. Based on the above research, optimizing the interlayer spacing can facilitate the rapid transfer of NH_4_^+^ and improve the kinetics of the electrochemical reaction.

#### Porous architecture regulation

A well-developed porous structure increases the surface area, providing more active sites for electrochemical reactions, enhancing charge storage capacity and improving reaction kinetics [74]. Due to van der Waals forces between the layers of 2D nanomaterials, they often suffer from self-stacking problems, resulting in reduced specific surface area and fewer active sites for ion interaction, as well as poor ion transport kinetics. Asymmetric carbon nanoparticles lack pores or have a single pore structure, which limits the utilization of their internal matrix and often causes blockage, resulting in a poor ion diffusion effect. Ma *et al*. [75] synthesized asymmetric carbon nanoparticles with macro/mesopores, overcoming the defects of low ion conductivity and poor mass transfer. By regulating the porous structure of 2D materials, these problems can be effectively mitigated, and the capacity of batteries and capacitors can be further enhanced with a smaller amount of active material load [76].

Ozdoganlar *et al*. [77] introduced MXene into a freeze-dried porous silica skeleton (MX-PS) with open hierarchical porosity and a controlled porosity distribution by using capillary flow. Through a series of experimental studies, they found that the material exhibited the highest conductivity at an MXene concentration of 180 mg mL^−1^ and a framework porosity of 60%. Sandwich supercapacitors with MX-PS as electrodes showed excellent area capacitance (7.24 F cm^−2^) and energy density (0.32 mWh cm^−2^). This is mainly attributed to the 3D porous structure, which provides rapid ion and charge transfer channels for superior electrochemical performance. Pollet and collaborators prepared a porous prism architecture of lithium-rich layered oxide materials [78]. The porous morphology enhances the intercalation and extraction of Li^+^, resulting in a high initial discharge capacity of 295.3 mAh g^−1^ at 0.1 C and excellent rate capability. Additionally, Ren and his co-workers [79] synthesized 2D porous Ti_3_C_2_T*_x_* MXene by partial oxidation and chemical etching in aqueous solution at room temperature. The prepared porous Ti_3_C_2_T*_x_* MXene has a larger specific surface area and a more open structure than the original Ti_3_C_2_T*_x_* MXene, which is conducive to ion diffusion and improves electrochemical kinetics. The porous structure can be constructed via various methods, such as the freeze-drying method [80], template method [81], gelation method [82] and etching method [83]. Although the application of this strategy in AAIBs is still limited, the materials prepared by this approach are expected to significantly enhance the performance of AAIBs.

### Surface chemistry regulation

#### Targeted surface termination regulation

In recent years, 2D MXene materials have demonstrated significant potential in various fields due to their high electrical conductivity, chemical stability, hydrophilicity, diversity and adjustable surface chemistry [84]. The general formula for MXenes is M_*n*+1_X_*n*_T*_x_*, where M represents an early transition metal (such as Ti, Mo or Cr), X represents carbon and/or nitrogen, *n* is typically an integer between 1 and 4, and T*_x_* denotes surface terminations, such as hydroxides, oxygen or fluorine. These surface terminations contribute to the hydrophilicity of MXenes and render them unique properties [85,86].

Notably, researchers have shown that MXene surfaces with a higher concentration of –O functional groups can achieve higher energy density [87,88] (Fig. 5b). Sun and his team [61] found through theoretical calculations that V_2_CO_2_ has the maximum work function, and V_2_CT*_x_* MXene exhibits a pseudocapacitive nature for storing NH_4_^+^, providing a remarkable specific capacity of 115.9 mAh g^−1^ at 1 A g^−1^. Molecular dynamics simulation results indicated that V_2_CT*_x_*with abundant –O terminations can achieve high capacity. Gao's group [89] successfully prepared hydroxylated Ti_3_C_2_ MXene (h-Ti_3_C_2_ MXene) with a carpet-like structure and more O-containing functional groups. This modification tailored the NH_4_^+^/H^+^ co-adsorption chemistry of the materials. The presence of these functional groups improved the performance of thick electrodes, which typically suffer from the stacking property of 2D Ti_3_C_2_T*_x_* MXene, and enhanced the proximity of H^+^ and NH_4_^+^ to the electrode material. As a result, h-Ti_3_C_2_ MXene with NH_4_^+^/H^+^ co-adsorption exhibited a high capacitance of 274.3 F g^−1^ at a current density of 1.0 A g^−1^. However, O-rich MXenes are prone to oxidation, which can reduce their electrical conductivity. Several measures can be taken during preparation and application to mitigate oxidation. During preparation, antioxidants can protect MXene surfaces from exposure to air, and controlling the ambient oxygen concentration can also help [90]. During application, careful preservation of MXenes to avoid exposure to oxygen is crucial. These strategies can help maintain the desirable properties of O-rich MXenes while reducing the risk of oxidation.

#### Surface defect regulation

Surface defect engineering is a technique that optimizes the performance of materials by introducing controlled defects on their surfaces. By fine-tuning the surface structure and electronic states, this method significantly enhances ion conductivity, promotes ion transport, increases interfacial reactivity and improves cycling stability. In particular, surface defects can create additional active sites that enhance the interaction with the electrolyte and increase the reaction rate of the electrode materials. In addition, these defects can reduce energy loss of batteries during the charging and discharging processes, which improves overall efficiency, thereby providing robust technical support for the development of high-performance batteries [91].

MXenes produced by acid etching inevitably form metal vacancies or vacancy clusters during the etching process [76] (Fig. 5b). Sun *et al*. demonstrated that the presence of these vacancies significantly affects the electrochemical performance of MXenes [92]. The presence of vacancies can be used as a potential trap to enhance the adsorption of Li^+^ in the region near the defect, and the individual effect is minimal on the charge and discharge rate of Mo_2_C MXene. The reduction in the molecular mass of the host material due to these defects results in an increase in the theoretical specific capacity. Specifically, the theoretical specific capacity of the Mo_2_C-VMo model increased to 542 mAh·g^−1^. Similarly, Zhou's group [93] enhanced nitrogen-doped carbon by incorporating additional phosphorus doping, which created abundant pores and edge defects. This modification resulted in a high proportion of active N, enhancing conductive networks, accelerating the charge transfer and K^+^ diffusion kinetics, and exhibiting a high reversible capacity of 265.8 mAh g^−1^ after 2000 cycles at 1.0 A g^−1^. Wang’s group [58] prepared oxygen vacancy-enriched Bi_2_SeO_5_ nanosheets, which exhibited a remarkable reversible capacity of 341.0 mAh g^−1^ at a current density of 0.3 A g^−1^ in 1 M NH_4_Cl electrolyte, coupled with an outstanding capacity retention of 86.7% following 7000 cycles at 3 A g^−1^, demonstrating excellent NH_4_^+^ storage performance.

### Crystal water adjustment

NH_4_^+^ typically forms hydrogen bonds with materials, enhancing their storage capacity. The formation of hydrogen bonds between NH_4_^+^ and the material's interlayer crystal water is possible, which implies that controlling the interlayer crystal water content markedly enhances the storage performance of NH_4_^+^ [68]. Additionally, interlayer crystal water can increase the interlayer spacing and improve the structural stability of layered compounds, facilitating the reversible insertion/extraction of ions (Fig. 5c) [94].

As previously mentioned, Hu *et al*. [44] highlighted the beneficial role of crystal water in their study of VOPO_4_·2H_2_O. Using DFT, they created a computational model based on the hypothesis of NH_4_^+^ substitution of crystal water, revealing that the crystal water molecules between the VOPO_4_ layers play a crucial role in the NH_4_^+^ insertion process. Following this, the VOPO_4_·2H_2_O precursor was then annealed at different temperatures and subjected to electrochemical tests. The results showed that as the crystal water content decreased, the discharge plateau gradually disappeared, and the capacity decreased significantly, highlighting the importance of crystal water in VOPO_4_·2H_2_O. A similar enhancement effect of crystal water was observed in V_2_O_5_. Chen's group [55] studied the electrochemical properties of bi-layered V_2_O_5_ and annealed V_2_O_5_ at different temperatures, confirming the effect of crystal water on the material properties. These results clearly indicate that adjusting the content of crystal water between the layers of 2D materials can significantly facilitate NH_4_^+^ storage.

### Amorphous structure regulation

The disordered structure and abundant defects in amorphous materials significantly enhance ion diffusion through their lattice. These materials typically have numerous active sites, which facilitate ion intercalation and improve the capacity [95]. Liu *et al*. [37] prepared electrochemically activated Co-Ni double hydroxide (E-CoNi DH) for use as the anode material in AAIBs. Electrical activation broke the O–H bonds, converting some of the metal–O–H bonds to metal–O bonds and creating more H defects. This process resulted in a high degree of disorder and numerous crystal defects in the material. Electrochemical tests in various electrolytes showed that the E-CoNi DH electrode had a superior discharge capacity of 202.4 mAh g^−1^ at 0.6 A g^−1^ in NH_4_Ac electrolyte, surpassing the discharge capacities in KAc (168.5 mAh g^−1^) and NaAc (174 mAh g^−1^) electrolytes. The electrode also exhibited good rate performance, highlighting the superiority of NH_4_^+^ energy storage.

Similarly, Mn_3_Al_1_-LDH changed from its initial crystalline phase to an amorphous phase during the charge/discharge process, and maintained this amorphous phase well in the subsequent cycle [45]. The Mn_3_Al_1_ LDH electrode exhibited a high discharge capacity of 183.7 mAh g^−1^ at 0.1 A g^−1^. Due to the presence of numerous defects, the porous and amorphous MP-20 had an even higher capacity of up to 299.6 mAh g^−1^, and excellent rate performance [38]. These studies demonstrate that the amorphous structure and defects significantly enhance the electrochemical properties of the materials. Therefore, the creation of amorphous structures with numerous active sites is a promising strategy for the development of high-performance AAIBs (Fig. 5d).

### Construction of heterostructures

Heterostructures offer significant advantages in electrochemical applications through their interfacial effects and material synergies. These structures can facilitate the acceleration of electron transport through the internal electric field at the heterogeneous interface, promote rapid ion insertion and mitigate volume expansion during cycling through enhanced ion diffusion and synergistic effects [96,97]. The main methods for constructing heterostructures are the surface self-assembly strategy and the *in-situ* derivation strategy, as illustrated in Fig. 5e and f.

#### Surface self-assembly strategy

Heterostructures based on surface self-assembly strategies are formed by a spontaneous molecular or nanoscale assembly process on material surfaces. This approach exploits intermolecular interactions, such as van der Waals forces, hydrogen bonds, electrostatic interactions and π-π interactions, to guide the self-assembly of materials into ordered structures with specific functions on the surface [98–100].

Liu's group [59] developed a core-shell structured VO*_x_*@PPy anode using electrochemical methods for AAIBs. The interaction between nitrogen atoms in PPy and vanadium cations in VO*_x_* enhances the protonation level of PPy, reducing the vanadium in the composite and thereby improving the conductivity of the composite. This enhancement allows VO*_x_*@PPy to achieve a high specific capacity of 195.36 mAh g^−1^ at 0.2 A g^−1^. Additionally, the PPy coating effectively inhibits the dissolution of VO*_x_*, resulting in good cyclic stability (85% capacity retention after 2000 cycles for VO*_x_*@PPy compared to 51% capacity retention after 100 cycles for VO*_x_*). The composite showed enhanced electrochemical performance compared to VO*_x_* and PPy.

To address the low conductivity and dissolution problems of PTCDA during NH_4_^+^ insertion/extraction processes, Gao's group [39] introduced Ti_3_C_2_T*_x_* MXene, which has high conductivity and polar surface terminations, as the conductive skeleton for PTCDA through simple solution mixing and extraction filtration. The incorporation of Ti_3_C_2_T*_x_* MXene significantly improved the stability and conductivity of PTCDA. Compared to pure PTCDA, the composite material exhibited superior electrochemical properties. By constructing a heterogeneous structure, the combined advantages of multiple materials can be fully utilized, significantly enhancing the performance of the material (Fig. 5e).

#### *In-situ* derivation strategy

Heterostructures based on the *in-situ* derivation strategy are constructed directly during the material formation process. This approach typically involves the introduction of a precursor or template into the raw material, which is then transformed into a heterogeneous structure through a chemical reaction or physical process under specific conditions (Fig. 5f).

Due to the high conductivity of VS_2_ and the stability of metal oxides, Du's group [36] employed an *in-situ* electrochemical method to induce the formation of a heterostructured VS_2_/VO*_x_* material. In this process, fluffy VO*_x_* nanosheets were homogeneously grown on VS_2_, creating an interwoven porous electrode structure. Compared to pure VS_2_, the NH_4_^+^ storage behavior of the VS_2_/VO*_x_* heterostructure was significantly improved, achieving a capacity of 150 mAh g^−1^. At a high current density of 5 A g^−1^, the VS_2_/VO*_x_* heterostructure provided a specific capacity of 100 mAh g^−1^, whereas the pure VS_2_ electrode provided only 20 mAh g^−1^. Due to the high reactivity of the metal surface of MXenes, they are prone to oxidation in air, forming the corresponding oxides, which can alter their properties [101]. Yuan and his co-workers [102] synthesized layered accordion-like TiO_2_/Ti_3_C_2_ nano-hybrids through a scalable hydration process, where nano-sized TiO_2_ is uniformly decorated on the surface of Ti_3_C_2_. Due to the synergistic effects between TiO_2_ and Ti_3_C_2_, the resulting material exhibits enlarged interlayer spacing, facilitating ion insertion/extraction, and demonstrating enhanced capacity. It is promising to prepare MXene-based composites through *in-situ* oxidation or sulfidation to enhance the performance of battery systems.

## SUMMARY AND PERSPECTIVES

**Figure 6.**
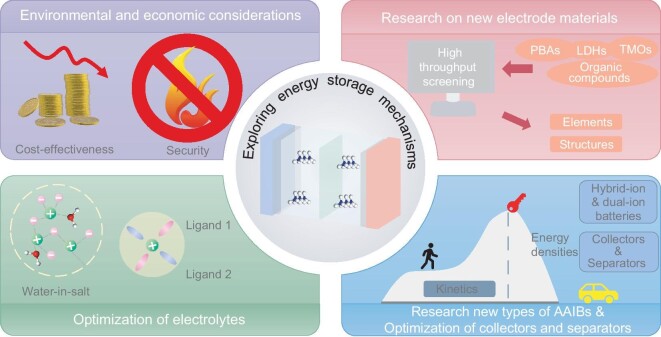
Prospects for promoting the development of AAIBs.

Many 2D materials have demonstrated excellent electrochemical properties in AAIBs due to their large diffusion channels and easily adjustable interlayer spacing. We have summarized various NH_4_^+^ storage mechanisms in 2D materials that contribute to their outstanding electrochemical performance. However, the small interlayer spacing, re-stacking tendencies and surface defects of 2D electrode materials can lead to instability after numerous cycles, significantly affecting their electrochemical performance. The modification strategies discussed above are anticipated to mitigate these issues and enhance the NH_4_^+^ storage properties of 2D materials. Despite significant progress, the transition of AAIBs from laboratory research to industrial applications is hampered by their low energy density. Furthermore, current research on AAIBs is still in its infancy, and significant efforts are needed to promote large-scale application. Additionally, AAIBs encounter other challenges, such as poor electrochemical stability and a complex, poorly understood energy storage mechanism. In the subsequent sections, we will explore perspectives aimed at advancing the development of AAIBs (Fig. 6).

### Environmental and economic considerations

All research efforts aim to achieve practical applications on an industrial scale. During the optimization process of materials and electrolytes, it is crucial to conduct a comprehensive assessment of cost effectiveness and environmental impact. 2D materials such as graphene stand out in terms of sustainability due to their exceptional physical properties and light weight. The preparation of 2D materials such as TMDs often involves the use of hazardous chemicals or energy-intensive processes; therefore, developing more environmentally sustainable production methods is required. Waste management practices should prioritize environmentally friendly approaches, including the recovery of sulfur components. Moreover, the relatively low consumption of 2D materials during the electrode manufacturing process facilitates easier recycling. The selection of raw materials should focus on those that are economically viable and accessible. Safety considerations are also paramount. For example, the use of ammonium salt electrolytes carries inherent risks due to their tendency to release NH_3_ gas at elevated temperatures. When this gas accumulates to a certain level, it can cause the battery to swell and, under certain conditions, potentially explode, posing a serious safety hazard to the battery system. Consequently, it is necessary to explore optimization methodologies that prioritize affordability, environmental friendliness, convenience and safety in both preparation and implementation.

### Research on new 2D electrode materials

The pivotal role of electrode materials in battery performance is underscored by their operational principles and structural composition. Currently, the selection of 2D electrode materials is mainly focused on TMOs, LDHs, MXenes and organic compounds. For TMOs, the exploration of new transition elements is crucial. Computational chemistry tools allow researchers to identify elements and structures suitable for NH_4_^+^ storage or to modify existing structures to enhance performance. In the case of MXenes, investigating new materials with novel transition elements or those that are resistant to dissolution in aqueous electrolytes could lead to significant advancements. Additionally, exploring the potential of NH_4_^+^ storage in various LDH systems presents promising opportunities. Research on organic compounds has predominantly focused on carbonyl-containing compounds. Expanding this research to include amine-containing compounds could provide new insights and possibilities. While most research on battery electrodes has concentrated on cathode materials, anode materials have received comparatively less attention. It is therefore imperative to broaden investigations into anode materials that are compatible with the identified cathode materials to achieve balanced and efficient battery systems. In the study of materials, it is essential to consider the feasibility of large-scale production. Currently, the large-scale implementation of 2D materials in AAIBs faces several critical challenges primarily related to cost and integration. Key issues include material cost, recycling expenses, material quality, consistency and market acceptance. Overcoming these challenges will require interdisciplinary collaboration and a commitment to in-depth research and technology optimization, highlighting the urgent need for further investigation and innovation in this field.

### Optimization of electrolytes

In addition to materials research, optimizing the electrolyte plays a crucial role in enhancing battery performance, particularly by accelerating rapid ion transfer. In the field of AAIBs, common electrolytes include mainly CH_3_COONH_4_, (NH_4_)_2_SO_4_ and NH_4_Cl, which have a relatively narrow operating window, thus limiting the capacity of the battery. Additionally, there are only a few types of electrolytes currently used, highlighting the need for further development of novel electrolytes to broaden the range of applications and improve performance. ‘Water-in-salt’ (WIS) electrolytes can reduce water activity, extend the voltage window and improve battery performance compared to their dilute counterparts under similar test conditions. Currently, WIS electrolytes, mainly CH_3_COONH_4_ and NH_4_TFSI, are under exploration, and there is significant potential to investigate other types of WIS electrolytes. Furthermore, electrolyte additives have proven to be effective in enhancing battery performance as they adjust the solvation structure, mitigate hydrolysis reactions and reduce electrode dissolution. However, research specific to electrolyte additives for AAIBs remains limited. By leveraging knowledge from other battery systems, new additives tailored to AAIBs can be explored to improve overall battery efficiency and stability.

### Optimization of current collectors and separators

Improving the materials, structures and performance of current collectors and separators is crucial for improving the overall performance of batteries, including conductivity, ion transport, mechanical stability and cycle life. Currently, titanium mesh and copper foil are widely used as single metal current collectors, with limited research into composite material alternatives. The introduction of composite materials such as carbon nanotubes, graphene and conductive polymers as coatings on metal surfaces can significantly enhance the conductivity, chemical stability and mechanical strength of current collectors. This approach promises to improve cycle life and energy density by preventing deformation and damage during charge/discharge cycles. In addition to material selection, choosing current collectors with high mechanical strength and excellent thermal stability is essential to ensuring durability under various operating conditions. For polyolefin separators such as polypropylene and polyethylene, which offer good chemical stability and mechanical strength but lower ion conductivity, modifications such as coating with inorganic nanomaterials (e.g. SiO_2_, Al_2_O_3_) or conductive polymers can enhance their thermal stability and ion transport capabilities. Optimizing the pore structure of separators further enhances the rate performance and capacity of batteries. By advancing current collector and separator technologies through these strategies, we can pave the way for improved battery efficiency and reliability in various applications.

### In-depth understanding of the NH_4_^+^ storage mechanism

A thorough understanding of the NH_4_^+^ storage mechanism in materials is essential for the design and refinement of new electrode materials. While the existing mechanisms for 2D electrode materials have been summarized, our current understanding remains incomplete. The characterization methods and theoretical calculation techniques used to study these mechanisms are relatively straightforward and often rely on *ex-situ* technologies, which may not accurately capture real-time changes in the morphology and structure of 2D electrode materials during charge/discharge processes. To overcome these limitations, employing *in-situ* testing technologies can offer clearer and more accurate insights into material transformations. Additionally, advanced computational methods such as high-throughput screening and machine learning can accelerate experimental processes and improve efficiency. These approaches promise deeper insights into NH_4_^+^ storage mechanisms, ultimately guiding the development of high-performance 2D electrode materials for AAIBs.

### Development of new types for aqueous ammonium-based batteries

The emergence of hybrid batteries, including dual-ion batteries, represents a notable advance in battery technology. These innovative energy storage systems use multiple charge carriers to potentially offer higher energy densities and operating voltages compared to traditional battery types. Hybrid batteries combine the strengths of different technologies to optimize performance, often resulting in more efficient and longer-lasting energy storage solutions. Additionally, flexible batteries are gaining attention due to their potential application in wearable electronics and other flexible device technologies. Their ability to conform to various shapes and sizes makes them ideal for integration into new and diverse electronic devices, opening up opportunities for more innovative and user-friendly products. However, research into ammonium-based hybrid or dual-ion batteries, and flexible AAIBs, remains limited. Increased research efforts could lead to significant breakthroughs, accelerating the progress of AAIB technology and expanding the range of applications for hybrid and flexible batteries.
